# *De novo* heterozygous pathogenic *FBN1* variant in an autopsy case of multiple aneurysms and right renal artery dissection: a case report

**DOI:** 10.3389/fcvm.2023.1170460

**Published:** 2023-06-12

**Authors:** Taylor MacGowan, Taylor McClinchey, Vibhu Parcha, Matteo Vatta, Silvio Litovsky, Pankaj Arora, Paul V. Benson

**Affiliations:** ^1^ Department of Pathology, University of Alabama at Birmingham, Birmingham, AL, United States; ^2^ Tulane University Pathologists’ Assistant Program, Tulane University, New Orleans, LA, United States; ^3^ Department of Genetics, University of Alabama at Birmingham, Birmingham, AL, United States; ^4^ Department of Medicine, Division of Cardiovascular Disease, University of Alabama at Birmingham, Birmingham, AL, United States; ^5^ Invitae Corporation, San Francisco, CA, United States

**Keywords:** case report, *FBN1*, Marfan, dissection, aneurysm

## Abstract

**Background:**

Marfan syndrome is a potentially fatal inherited autosomal dominant condition impacting the cardiovascular and the skeletal system with an estimated 25% cases caused by sporadic genetic variations. Given the genetic inheritance pattern, an autopsy of probands with Marfan syndrome–associated mortality is critical to establish the phenotypic expression and clinical implications of the particular genetic variant, especially for first-degree relatives. We present the findings of a Marfan syndrome proband decedent presenting with sudden onset abdominal pain and unexplained retroperitoneal abdominal hemorrhage.

**Methods:**

An autopsy was performed to inform the blood relatives of the phenotypic expression and penetrance of the potentially heritable condition. A clinical laboratory improvement amendment (CLIA)-certified clinical grade genetic sequencing was performed to identify pathogenic variants in genes associated with aortopathy.

**Results:**

The autopsy showed intra-abdominal and retroperitoneal hemorrhage due to infarction of the right kidney caused by dissection of the right renal artery. Genetic testing identified a heterozygous pathogenic *FBN1* gene variant. The specific variant is *FBN1* NM_000138.4 c.2953G > A p.(Gly985Arg).

**Conclusions:**

We report a case of a previously undiagnosed Marfan syndrome death due to a *de novo FBN1* variant, c.2953G > A.

## 1. Introduction

A 48-year-old Caucasian male presented with severe flank pain. Death occurred on hospital day 4 with right renal artery dissection, right renal infarction, and hemorrhage revealed at autopsy. Sequence analysis and deletion/duplication testing of 29 genes associated with hereditary aortopathy conditions identified a heterozygous pathogenic variant, *FBN1* c.2953G > A, in the decedent. This report of a *de novo FBN1* pathogenic variant demonstrates that Marfan syndrome may present with diffuse and potentially fatal involvement of the systemic vasculature.

## 2. Case description

**Figure 1:**
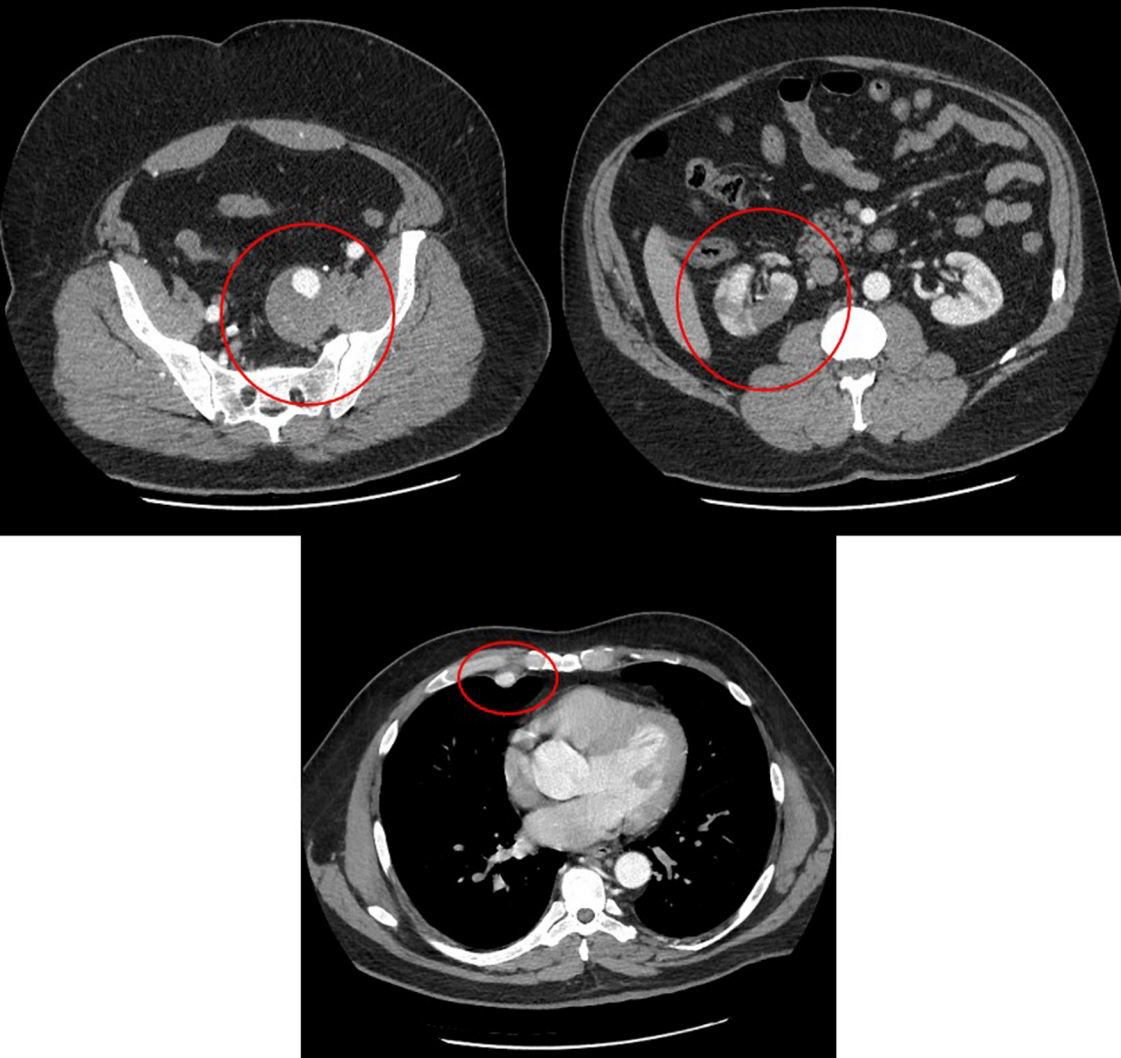
Computed tomography angiogram showing right renal infarction (right panel), left internal iliac artery aneurysm (left panel), and right internal mammary artery aneurysm (bottom panel).

A 48-year-old Caucasian male presented to our hospital for severe right flank pain. A computerized tomography angiography scan of the abdomen demonstrated a right renal infarct (Figure 1) and was started on intravenous heparin. The etiological evaluation of the source of a possible embolism was pursued. No intra-cardiac thrombus was noted on the transesophageal echocardiogram. The patient was also noted to have a 5.5 cm × 5.1 cm left internal iliac artery aneurysm with no aneurysmal leakage or dissection, and a vascular surgeon was consulted (Figure 1). Imaging was also notable for a right brachiocephalic artery aneurysm. With the growing concern for a systemic connective tissue disease, transfer to a tertiary healthcare center was sought. The transfer was not possible due to diversion at tertiary healthcare centers. On day 4 of admission, the patient became hypotensive with worsening right flank pain and radiographic evidence of a large right retroperitoneal hemorrhage precipitating blood transfusion. His hospital course was complicated by worsening mental status and escalating oxygen requirements, which prompted intubation. Subsequent to intubation, the patient became bradycardic, followed immediately by pulseless electrical activity cardiac arrest. Despite resuscitative attempts, and transfusion of eight units of packed red blood cells, the patient died on hospital day 4 (Table 1).

**Table 1:** A timeline with relevant data from the episode of care.

Hospital admission day	The patient presented to our emergency department with acute right flank pain. CT angiogram showed right renal infarction and multiple aneurysms. Renal infarction was considered to be embolic. He was treated with IV heparin. Creatinine: 1.67.
Hospital day 1	No complaints and controlled flank pain with opiates. Transthoracic echocardiogram EF 55%, no wall motion abnormalities, and mild aortic root and ascending aortic dilation.
Hospital day 2	Transesophageal echocardiogram was performed and was normal without thrombus. The patient was awake and alert, with flank pain well controlled. Plans considered to transfer the patient to a tertiary care facility for surgical repair of the right renal artery.
Hospital day 3	Continued mild right flank pain managed with opiates. Creatinine: 2.1.
Hospital day 4	Became hypotensive with worsening right flank pain. Large right retroperitoneal hemorrhage on CT. Worsening mental status and low oxygenation preceded intubation. The patient received eight units packed red blood cells (PRBC) and platelets with plasma. Bradycardia followed intubation, immediately followed by PEA and ventricular fibrillation. Advanced cardiac life support (ACLS) was started, and the patient was defibrillated multiple times for ventricular fibrillation, but pulse was never restored.

## 3. Diagnostic assessment, details on the therapeutic intervention, follow-up, and outcomes

A comprehensive autopsy was performed at the request of the clinicians and the family. An autopsy consent was obtained from the next of kin for diagnostic, teaching, and research use. A standard Y-shaped incision and biparietal intermastoid incision were performed. Following an *in situ* examination of the organs, the Letulle autopsy method was used to examine the organs of the torso and pelvis. Histologic sections of the heart, lungs, liver, kidneys, adrenal glands, thyroid gland, pancreas, brain, colon, aorta, right internal mammary artery, right renal artery, left renal artery, right subclavian artery, right coronary artery and left anterior descending coronary artery, left internal iliac artery, and subcarinal lymph nodes were examined. Verhoeff–van Gieson elastin stain (VVG) was performed on the right internal mammary artery, right renal artery, left renal artery, distal right renal artery, and aorta. Heart blood (4 ml) in an EDTA tube was sent to Invitae Corporation, San Francisco, California, for sequencing variations of 29 different genes (Supplementary Appendix S1). The external examination at autopsy was unremarkable. The decedent was 72 in. (6 ft) and 287 lbs (body mass index 38.9 kg/m^2^). The body showed evidence of medical intervention including burns from resuscitation attempts. The peritoneal cavity contained 500 ml of hemoperitoneum and abundant right retroperitoneal hemorrhage extending from the right hemipelvis to the right hemidiaphragm measuring 26 cm in maximum dimension. The right renal artery showed a dissection, 3 cm distal to the origin from the aorta with a focal sub-1 cm intimal defect noted just proximal to the first bifurcation of the right renal artery (Figure 1). The dissection of the right renal artery focally compressed the true lumen of the right renal artery with a thrombosis of the false lumen (Figure 1). There was diffuse marked hemorrhagic infarction of the right kidney. The left renal artery appeared grossly unremarkable, and the left kidney was pale. Multiple non-ruptured arterial aneurysms were present, such as a 2.0 cm  × 2.0 cm aneurysm of the proximal right internal mammary artery; a 6.0 cm  × 3.0 cm × 3.0 cm aneurysm of the right subclavian artery, 2 cm distal to the bifurcation of the brachiocephalic artery; and a 7.0 cm  × 5.0 cm  × 5.0 cm aneurysm of the left internal iliac artery, 2.5 cm distal to the bifurcation of the left common iliac artery. Changes of hypertensive cardiovascular disease were present including cardiomegaly (680 g), mild nephrosclerosis, and mild non-obstructive patchy calcification of the coronary arteries. The lungs showed pulmonary edema and mild emphysematous change. The colon showed a 1.7 cm pedunculated adenomatous polyp 15 cm distal to the ileocecal valve. There was mild cavum septum pellucidum of the brain, mild follicular hyperplasia of the thyroid gland, and mild steatosis of the liver.

**Figure 2:**
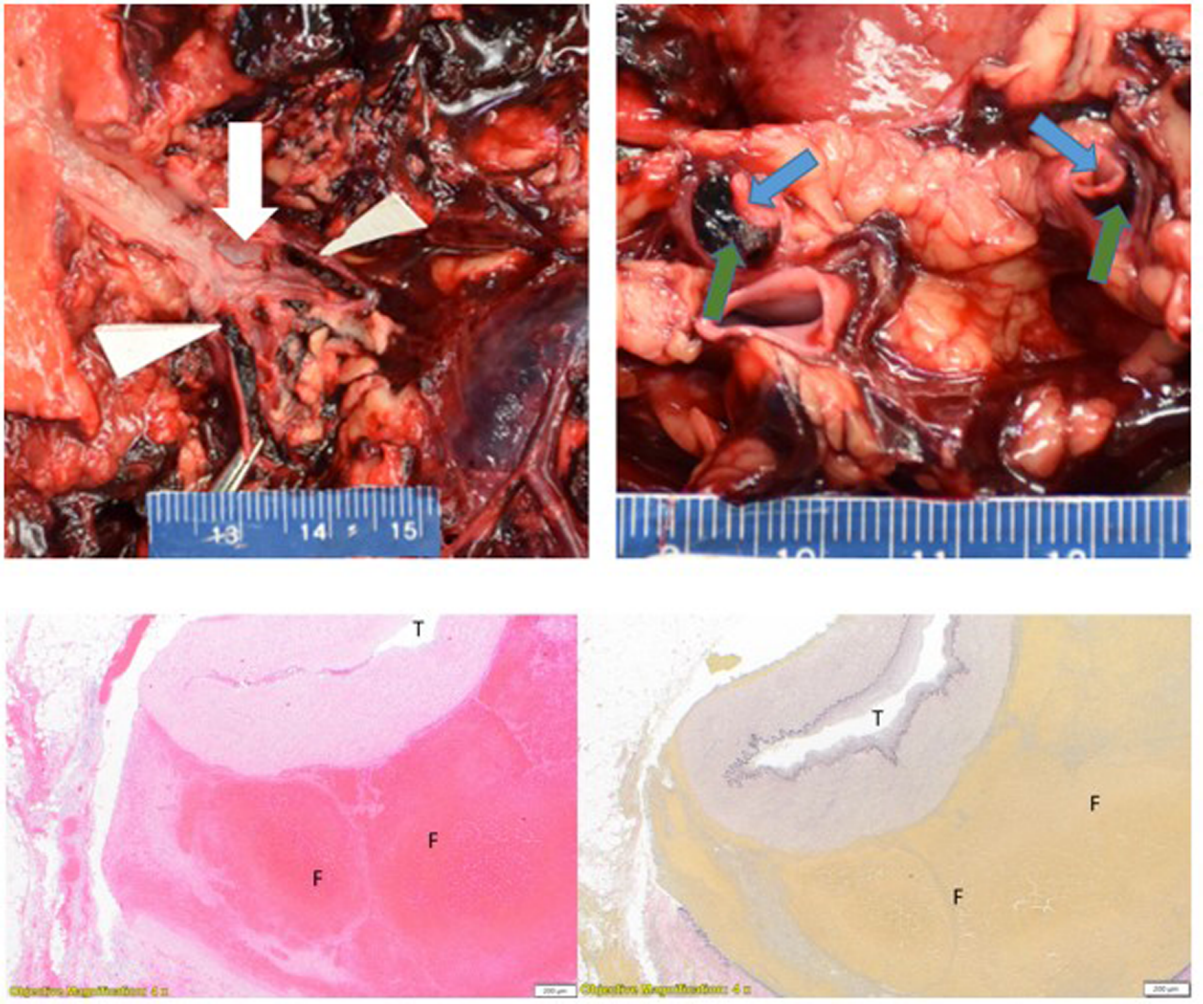
Left upper panel: right renal artery dissection with intimal defect (white arrow) and dissection distal to bifurcation (white triangles). Right upper panel: longitudinal section of a bifurcated right renal artery with false lumen (green arrows) and true lumen (blue arrows). Left lower panel: right renal artery dissection. (T = true lumen, F = false lumen with thrombus) H&E stain 4×. Right lower panel: (T = true lumen, F = false lumen with thrombus) VVG stain 4×.

Histologic examination of the right renal artery showed acute dissection with fibrolamellar elastin fragmentation and loss confirmed by VVG elastin stain. Organizing thrombus was present in the false lumen of the right renal artery dissection (Figure 2). The right kidney showed cortical infarction with abundant necrosis. The left renal artery showed slight focal medial dissection and focal intramural organizing hematoma with fibrolamellar elastin fragmentation and loss confirmed by VVG elastin stain. The left kidney showed moderate nephrosclerosis with arteriosclerosis, scattered sclerotic glomeruli, and arteriolar sclerosis with occasional hyaline change. The right internal mammary artery aneurysm showed organizing thrombus in the aneurysm with fibrolamellar elastin fragmentation and loss confirmed by VVG elastin stain. The right subclavian artery aneurysm showed elastin degradation with intramural organized hematoma. The left internal iliac artery aneurysm showed moderate atherosclerosis with degradation of the elastic lamina and organizing thrombus. Sections of the coronary artery showed focal intramural non-occlusive hemorrhage and moderate degradation of the internal elastic lamina. The aorta showed moderate fibrolamellar elastin fragmentation with mild to moderate mucopolysaccharide deposition and multifocal cystic medial necrosis. The heart showed diffuse myocyte hypertrophy and mild perivascular fibrosis. There was mild emphysematous change of the lungs and mild steatosis of the liver.

The cause of death was determined to be hemorrhagic shock due to infarction of the right kidney due to occlusive dissection of the right renal artery due to the *FBN1* gene variant.

Sequence analysis and deletion/duplication testing of 29 genes showed the decedent to be heterozygous for a pathogenic variant of the *FNB1* gene, specifically *FBN1* NM_000138.4 c.2953G > A p.(Gly985Arg). Although no paternity testing was performed, markers analyzed for quality control purposes were sufficient to support relatedness between the deceased and the parental samples.

**Figure 3:**
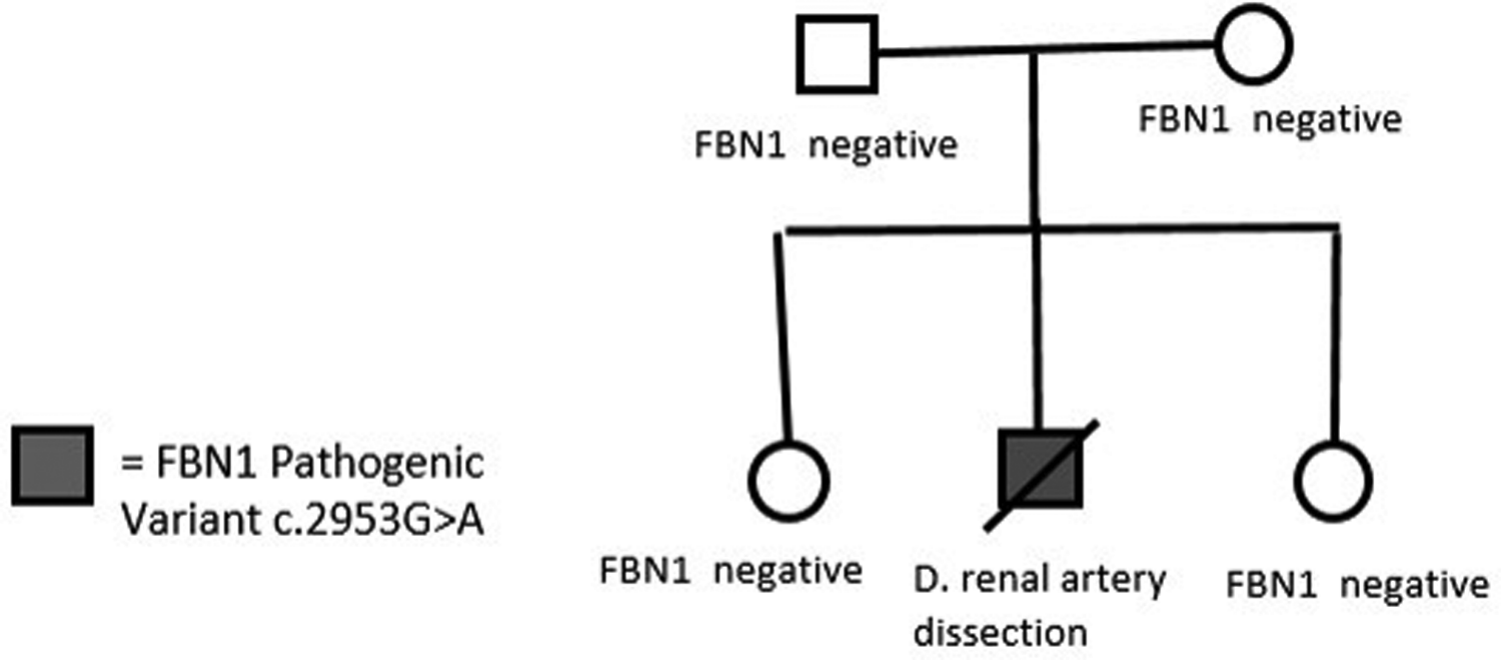
Pedigree with negative first-degree genetic testing results.

Clinical consultation was recommended to the family, and subsequent cascade screening of first-degree relatives was pursued at the University of Alabama at Birmingham Cardiovascular Genomics Clinic. Genetic testing did not identify the same variant or other pathogenic variants in *FBN1* in any of the parents and siblings (Figure 3). This established the *de novo* existence of the genetic variant in the deceased proband.

## 4. Discussion

Marfan syndrome is caused by an inherited defect in fibrillin-1, which is an extracellular glycoprotein found in the extracellular matrix of cells that controls the availability of free transforming growth factor (TGF)-beta (1). Fibrillin-1 is encoded by *FBN1*, and fibrillin-2 is encoded by *FBN2*. Pathogenic variants in *FBN1* are associated with Marfan syndrome (2). Fibrillin proteins form a mesh network around a central core of elastin, which, in turn, forms elastic fibers (3). Microfibrils are found throughout the body, especially in the tunica media of the aorta and lens (1). Defects in fibrillin lead to weakened aortic walls and skeletal abnormalities (2). Marfan syndrome is caused by an inherited defect in fibrillin-1 (2). There are two mechanisms in which pathogenic variants in *FBN1* may contribute to the phenotype. The first is abnormal protein expression leading to a dominant-negative effect when the mutated protein interacts with normal protein in the extracellular matrix, and the second is haploinsufficiency from a reduced amount of fibrillin-1 protein produced as a result of a null allele (4, 5, 6). This case showed multiple aneurysms throughout the systemic vasculature. An examination of these aneurysms showed focal evidence of previous dissection best seen with VVG elastin stain. An examination of the grossly non-dissected left renal artery showed foci of intramural, non-extensive dissection consistent with diffuse vascular deficiencies. This report of a *de novo FBN1* pathogenic variant (c.2953G > A) detected in a deceased proband demonstrates that Marfan syndrome may present with diffuse and potentially fatal involvement of the systemic vasculature.

## Data Availability

The original contributions presented in the study are included in the article, and further inquiries can be directed to the corresponding author.
